# Corticosteroid-Induced Psychosis: A Report of Three Cases

**DOI:** 10.7759/cureus.39221

**Published:** 2023-05-19

**Authors:** Anil K Bachu, Victoria Davis, Mouad Abdulrahim, Lauren Harbaugh, Sakshi Prasad, Vijaya Padma Kotapati, Sushma Srinivas

**Affiliations:** 1 Psychiatry and Behavioral Sciences, Baptist Health - University of Arkansas for Medical Sciences (UAMS), Little Rock, USA; 2 Psychiatry and Behavioral Sciences, Allegheny Health Network, Pittsburgh, USA; 3 Psychiatry and Behavioral Sciences, Psychiatry Residency Program, Baptist Health - University of Arkansas for Medical Sciences (UAMS), North Little Rock, USA; 4 Psychiatry, Arkansas College of Osteopathic Medicine, Fort Smith, USA; 5 Internal Medicine, National Pirogov Memorial Medical University, Vinnytsia, UKR; 6 Psychiatry, Psychiatry Residency Program, Baptist Health - University of Arkansas for Medical Sciences (UAMS), North Little Rock, USA

**Keywords:** case-series, schizophrenia, psychosis, corticosteroid psychosis, steroid use

## Abstract

Corticosteroids are commonly used for pain management and inflammatory conditions but can cause neuropsychiatric complications ranging from anxiety to severe mood and psychotic symptoms. These complications can occur shortly after steroid treatment begins or at any point during therapy, and even after treatment has stopped. We present three cases of corticosteroid-induced psychosis in patients being treated for pain. The mechanism behind these complications is not fully understood, but stress on the hypothalamic-pituitary-adrenal (HPA) axis is thought to play a role. Clinicians should be cautious and regularly evaluate patients to minimize the risk of complications. More research is needed to understand the underlying pathophysiology.

## Introduction

Corticosteroids have a broad spectrum of uses in pain management, chronic inflammatory conditions, and palliative care. However, it is known to induce neuropsychiatric side effects ranging from aggressive and violent behavior to benign mood changes to depression, hypomania, or frank psychosis [1]. Although uncommon, It is estimated to be seen in 5-18% of the patients put on corticosteroids [2]. Therefore, they are classified as a subtype of substance or medication-induced psychosis under the Diagnostic and Statistical Manual of Mental Disorders, Fifth Edition (DSM-5) [3]. Although the risk factors behind this remain elusive, it is often dose-dependent and seen at higher doses, according to The Boston Collaborative Drug Surveillance Program. They reported that psychiatric disturbances were seen in 18.6% of patients on > 80mg prednisone or methylprednisolone, 4.6% on 41- 80 mg/day, and 1.3% on <40 mg or less per day [4]. Although the exact cause remains unknown, stress induced by exogenous steroids on the hypothalamic-pituitary-adrenal (HPA) axis is the implied mechanism. The current evidence has shown that tapering or discontinuing the offending steroid is the first step to managing the symptoms in most cases. Still, if they persist, additional antipsychotic coverage may be indicated.

In this case series, we report three cases of corticosteroid-induced psychosis that we saw at our hospital in patients treated with corticosteroids for pain management (as an anti-inflammatory in one patient who suffered from chronic pain). 

## Case presentation

Case 1

A 72-year-old male was brought by his wife to the emergency department (ED) with complaints of bizarre behavior, flight of ideas, and pressured speech. While waiting in the ED, the patient became paranoid and verbally aggressive with staff and security and ran out of the hospital to try and get into the car to leave. He realized his wife had the keys and walked 18 miles back home. Once he reached home, he was met by the police, who had been called due to his wife’s concern. He agreed to come to the hospital for an evaluation when he presented to our service. Three days before this presentation, the patient had been evaluated at a nearby hospital for two weeks of poor sleep, pressured speech, and paranoia. Labs had been primarily within normal limits, and imaging revealed no acute intracranial process, so the patient had been discharged shortly after admission. After returning home, the patient exhibited symptoms of paranoia, hypervigilance, pressured speech, poor sleep, and psychomotor agitation and was thus brought to our hospital by this wife for the current presentation.

He had no past psychiatric history and had a medical history of arthritis. Three weeks prior, he had received a steroid injection in his elbow. He reported that shortly afterward, he felt “great!” He reported having more energy than he had in the past and reported increased goal-directed activities. Later, the patient became paranoid and exhibited intrusive and verbally aggressive behaviors. He became increasingly concerned and perseverative on the thought that his wife was suffering from dementia and took her to the hospital for an evaluation.

On initial presentation in the ED, the patient was hyperverbal, disorganized, restless, fidgety, and paranoid. He was also seen jumping up and down as if to exercise several times throughout the day. The patient also had intrusive behavior, repeatedly asking staff random questions. Labs performed during hospitalization were all within normal limits, including vitamin levels, urodynamic studies (UDS), complete blood count (CBC), basic metabolic panel (BMP), and thyroid-stimulating hormone (TSH).

The patient was started on 25 mg of quetiapine twice daily to target his mood and help with his sleep. On the second day of hospitalization, the patient reported some improvement in his sleep and his thoughts but reported some daytime somnolence. We switched the patient's quetiapine to 50 mg nightly to address the daytime somnolence and further help his sleep. On the third day of hospitalization, the patient reported improved sleep and noted that he could think “more clearly.” He was less tangential and could engage with interviews appropriately by answering questions directly. He was also significantly less paranoid and more alert to his surroundings. When asked about his concern for his wife possibly having dementia, the patient could rationalize his thought process better. He noted that she had some memory issues and wanted her to be evaluated. The patient was less pressured, and both son and wife felt comfortable discharging the patient home. The plan was made to discharge the patient on a seven-day course of 50 mg Seroquel®, and he was educated on the risks and benefits of further steroid use in the future. 

Case 2

A 23-year-old Caucasian male with a history of irritable bowel disease presented with signs and symptoms of catatonia and psychosis. Four days prior to this presentation to the psychiatric service, he had been hospitalized for an acute flare of ulcerative colitis and treated with IV steroids (IV methylprednisolone 40 mg thrice daily) for two days. Then he had been transitioned to prednisone tablet 40 mg daily for three days. On initial presentation to the psychiatric service, he exhibited overt mutism, blunted affect, and poor hygiene. Throughout his hospitalization, he also demonstrated other signs of catatonia, including waxy flexibility, echopraxia, and negativism. He also appeared grossly psychotic and was often seen responding to internal stimulation by exhibiting inappropriate periods of laughter. The patient was previously reported as having a similar brief episode of psychosis four to five months before this admission but had never been hospitalized for mental health issues or diagnosed with a psychiatric condition. 

While the patient was initially diagnosed with catatonia secondary to steroid-induced psychosis, based on his lack of clinical improvement despite abstinence from steroids, he was eventually diagnosed with schizophreniform disorder. His initial treatment consisted of a regimen of benzodiazepines to target his symptoms of catatonia. He was started on lorazepam 2 mg twice daily, which was increased to 2 mg in the morning, 1 mg at 1200 hours, and 2 mg in the evening over three days. The patient showed a slight improvement in his catatonic symptoms after being started on lorazepam. Still, his dosing had to be adjusted several times (ranging from 1.5 mg twice daily to 0.25 mg twice daily) due to concerns of oversedation. Ultimately, his symptoms of catatonia could be mostly quelled on 0.5 mg thrice daily due to oversedation. Electroconvulsive therapy was also considered a potential treatment option for the patient, but this was not feasible due to facility constraints and lack of availability.

He was not started on an antipsychotic on initial presentation due to concerns of potentially worsening his catatonic symptoms. However, as his symptoms of catatonia began to improve, he exhibited positive symptoms of psychosis, such as inappropriate laughter and thought-blocking, secondary to internal stimulation. Thus, he was started on risperidone 0.25 mg twice daily, which was increased to 1 mg twice daily over three days before being discontinued 17 days later due to lack of improvement. He was then started on olanzapine 2.5 mg twice daily, which was increased to 5 mg twice daily over five days before being discontinued on his 31st day of admission due to continued minimal improvement.

He was then started on clozapine 25 mg daily, which was titrated up to 25 mg thrice daily over seven days. The patient showed marked improvement in terms of positive symptoms. The clozapine was briefly held (24 hours) out of an abundance of caution for myocarditis as his absolute eosinophil count (cells/uL) rose from 400 to 700 over two days but it was resumed due to a lack of clinical symptoms corresponding with the diagnosis. However, the clozapine was discontinued 15 days after its initiation due to alanine transaminase (ALT) and aspartate aminotransferase (AST) elevation (115 and 75, respectively). He was then started on paliperidone 3 mg once a day, which was increased to 6 mg on day 48 of admission. The patient initially showed worsening positive symptoms of psychosis after being switched to paliperidone, but these gradually resolved over the two days following initiation. For his negative symptoms (anhedonia and avolition), he was started on modafinil 100 once a day on his 15th day of hospitalization, which was titrated up to 350 mg in the morning over 16 days; on his 27th day of hospitalization, he was also started on venlafaxine 75 mg in the morning which was increased to 225 mg over 18 days. Reduction of negative symptoms was achieved with this combination. The venlafaxine and modafinil were slowly tapered and discontinued as the patient approached discharge, and the negative symptoms remained resolved. The patient was discharged with paliperidone 6 mg once a day for her psychotic symptoms and lorazepam 0.5 mg thrice daily to prevent catatonia relapse.

Case 3

A 36-year-old female with a psychiatric history of depression and anxiety was admitted to the ED for acute psychosis. She had a prior history of steroid-induced psychosis with a similar presentation. She recently had an endoscopy and was started on a regimen of steroids. Since then, her behavior has been drastically changed. The patient had not been sleeping and made bizarre statements of “unlocking her brain” and discovering the “secret of life.” The patient was also delusional about being pregnant. Beta human chorionic gonadotropin (hCG) was negative on admission, and UDS was positive for tetrahydrocannabinol (THC). The patient's home medications of Celexa and Wellbutrin were discontinued. On initial evaluation, the patient was shown to be paranoid, grandiose, hyperverbal, unoriented to month, place, and situation, and demonstrated disorganized thought processes. Because of her inability to appreciate the decision to consent to admission, she was placed on a 72-hour hold and was granted 45-day treatment.

During her admission, she displayed bizarre and disorganized behavior, such as speaking in a made-up, fictitious language, walking around naked in the unit, becoming selectively mute, and voicing delusions of being pregnant, despite taking a pregnancy test which was revealed to be negative. She also has been very intrusive with staff members and disrupting the unit requiring multiple PRN (pro re nata) medications. She was started on risperidone 2 mg twice daily and clonazepam 1 mg twice daily but refused to take these medications along with her prior medication indicated for her underlying diagnosis of hypertension, hyperlipidemia, and diabetes. The patient was paranoid about taking the pills. Hence, Risperdal was discontinued, and the patient was started on haloperidol 2 mg twice daily liquid. The patient showed significant improvement in the thought process, speech, and symptoms of paranoia, a more linear thought process with less thought blocking. The patient did not exhibit any aggressive behaviors in the unit. Acute psychotic symptoms were resolved with this combination. Clonazepam was slowly tapered and discontinued as the patient approached discharge, and the acute psychosis remained resolved. She was restarted on home medication Citalopram upon discharge. The patient was discharged on haloperidol 2 mg at bedtime for her psychotic symptoms and citalopram 40 mg daily for depressive symptoms. 

## Discussion

Since the 1950s, synthetic steroids have been extensively utilized and are frequently linked to systemic benefits and side effects. They are commonly prescribed as anti-inflammatory/immunosuppressant medications-corticosteroids affect T cell-mediated inflammation by suppressing cytokines and impeding the immunostimulatory function of monocytes and macrophages. According to DSM-5, for a patient to be diagnosed with substance or medication-induced psychosis, such as steroid-induced psychosis, there must be a psychotic symptom often after exposure to the medication, causing significant functional impairment. Thus it is more of a diagnosis of exclusion after ruling out all other causes, such as organic psychiatric disorders, drug use, intoxication, metabolic disturbances, infections, and neoplasms [3].

The neuropsychiatric complications associated with steroid treatment often range from clinically significant anxiety and sleep disturbances to severe mood symptoms such as depression, hypomania, mania and psychotic disorders, cognitive impairment, delirium, and dementia [5]. It occurs shortly after administering steroids, typically a median of three to four days, but it can occur at any time during the therapy or even after treatment discontinuation [6].

The exact pathophysiology remains unclear, but the stress induced by the exogenous steroids on the HPA axis is the implied mechanism. It causes a decrease in corticotropin-releasing hormone (CRH) and adrenocorticotropic hormone (ACTH) levels and cortisol levels by preferential activation of the glucocorticoid receptors in the adrenal glands. This imbalance can trigger neurocognitive and emotional disturbances such as delirium, mania, and disturbances in mood [7,8]. Furthermore, in animal models, it was observed that corticosteroids cause an increase in tyrosine hydroxylase and, in turn, increase dopamine, which may induce psychosis [8]

In the cases in the current report, all three patients had a history of steroid use before the onset of the psychiatric symptoms. In all the cases, the patients have been prescribed steroids to manage chronic pain. All these patients typically presented with psychiatric symptoms shortly after the initiation of steroid treatment; they all showed an improvement in their symptoms after withdrawing the steroid while starting a course of antipsychotic medication, just as reported in the existing literature. Typically there is a higher chance of developing steroid-induced psychosis if they have a history of developing it, as we see in Case 3.

The most effective treatment recommended is tapering or discontinuation of the offending agent. Psychiatric symptoms are usually treated with first-generation antipsychotics such as haloperidol or second-generation antipsychotics such as risperidone, olanzapine, and quetiapine. Second generations are preferred due to less extrapyramidal side effects. These drugs act on the dopamine D2 receptors in the central nervous system that helps reverse steroid-induced psychosis. In addition, medications like lithium and antidepressants such as selective serotonin reuptake inhibitors (SSRIs) are often prescribed to treat mania and depression symptoms.

## Conclusions

Clinicians should exercise caution when prescribing corticosteroids, considering their potential neuropsychiatric side effects. Timely and appropriate evaluation is vital to minimize the risk of complications. Further research is necessary to better understand the underlying pathophysiology. Although there is a lack of extensive research in the field of steroid-induced psychosis due to its unpredictable nature, it is crucial to acknowledge its significance as it can be distressing and dangerous for patients. Diagnosis primarily involves excluding other potential causes, while prevention primarily focuses on minimizing dosages and avoiding unnecessary prolongation of treatment.
